# Recruitment of Vitronectin by Bacterial Pathogens: A Comprehensive Overview

**DOI:** 10.3390/microorganisms12071385

**Published:** 2024-07-08

**Authors:** Angelica Pellegrini, Giampiero Pietrocola

**Affiliations:** Biochemistry Unit, Department of Molecular Medicine, University of Pavia, Viale Taramelli 3/b, 27100 Pavia, Italy; angelica.pellegrini@unipv.it

**Keywords:** vitronectin, bacterial surface proteins, bacterial–host interaction, bacterial adhesion, bacterial immune evasion, human ligands

## Abstract

The key factor that enables pathogenic bacteria to establish successful infections lies largely in their ability to escape the host’s immune response and adhere to host surfaces. Vitronectin (Vn) is a multidomain glycoprotein ubiquitously present in blood and the extracellular matrix of several tissues, where it plays important roles as a regulator of membrane attack complex (MAC) formation and as a mediator of cell adhesion. Vn has emerged as an intriguing target for several microorganisms. Vn binding by bacterial receptors confers protection from lysis resulting from MAC deposition. Furthermore, through its Arg-Gly-Asp (RGD) motif, Vn can bind several host cell integrins. Therefore, Vn recruited to the bacterial cell functions as a molecular bridge between bacteria and host surfaces, where it triggers several host signaling events that could promote bacterial internalization. Each bacterium uses different receptors that recognize specific Vn domains. In this review, we update the current knowledge of Vn receptors of major bacterial pathogens, emphasizing the role they may play in the host upon Vn binding. Focusing on the structural properties of bacterial proteins, we provide details on the residues involved in their interaction with Vn. Furthermore, we discuss the possible involvement of Vn adsorption on biomaterials in promoting bacterial adhesion on abiotic surfaces and infection.

## 1. Introduction

### 1.1. Vitronectin Structure and Physio/Pathological Role in the Host

Vitronectin (Vn) is a multidomain glycoprotein of 75 kDa synthetized mostly in the liver and secreted in the plasma, produced to a lesser extent at the level of platelets and macrophages. After an initial synthesis as a single-chain protein, Vn undergoes a proteolytic cleavage at Arg^379^-Ala^380^ due to a Vn polymorphism (substitution of methionine to threonine at position 381 in the C-terminal domain), resulting in a disulfide-bonded two-chain form [1].

**Figure 1:**
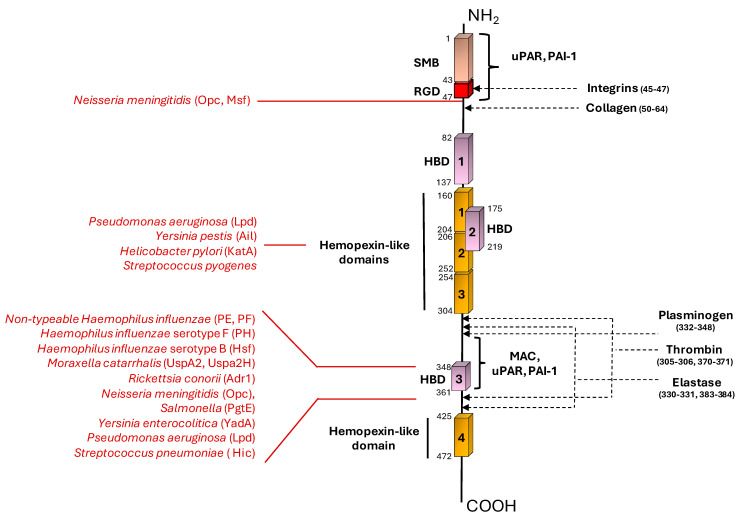
A schematic organization of vitronectin domains and ligand binding sites. A schematic representation of linear Vn structure showing the positions of Vn domains. Vn natural ligands are reported on the right (in black). Bacterial ligands are indicated on the left (in red). The N-terminal region of Vn contains a region homologous to somatomedin B (SMB), indicated in pink. The cell-binding motif RGD constitutes the integrin-binding domain, indicated in red. The three heparin-binding domains (HBD) are reported in purple (HBD-1, HBD-2, and HBD-3). The central and the C-terminal domains contain four regions homologous to hemopexin, the hemopexin-like domains, reported in orange. The C-terminal region is the one involved in binding by most of the ligands. The big majority of bacterial pathogens bind to C-terminal HBD-3. *N. meningitidis* is the only bacterial species that has been shown to bind to the N-terminal region of Vn. Full names and references of the various ligands are reported in the main text.

Vn is highly present in plasma (200–400 μg/mL), the extracellular matrix, and bone matrix, where it binds several structurally different molecules [2]. To do so, this protein is organized into a variety of distinct domains. The N-terminal domain contains a 44-residue region, identical to somatomedin B (SMB), which is henceforth referred to as the SMB domain. The SMB domain is crucial to wound healing, as it is also involved in Vn binding by the plasminogen activator inhibitor-1 (PAI-1) and the urokinase/plasminogen activator receptor (uPAR) [3,4,5]. This region is followed by the RGD domain that mediates Vn binding to integrins [6]. Following the RGD domain, there is the collagen-binding region followed by the first out of three heparin-binding domains, the HBD-1 [7]. Near the N-terminal domain, the central region comprises three hemopexin-like domains, including a highly charged sequence involved in heparin binding, which constitutes the second heparin-binding domain (HBD-2) [8,9]. The C-terminal region is the multi-functional portion that contains the third heparin-binding domain (HBD-3) [10] and the hemopexin domain 4. Moreover, this region constitutes a binding site for plasminogen [11], thrombin [12], elastase, [13] and a second binding site for PAI-1 and uPAR [4,5,12] (Figure 1).

Vn is a conformationally labile molecule, and this characteristic plays an important role in the regulation of protein function. Vn is normally present in blood in a native, inactive form. In the native conformation, different sensitive epitopes of the protein, such as the RGD integrin-binding domain, are not exposed. For this reason, blood Vn is commonly not an adhesive glycoprotein [6]. The in vivo activation of Vn occurs either in the presence of heparin or through Vn complexes’ formation, such as Vn-thrombin-antithrombin III, Vn-terminal complement proteins, or Vn-PAI-1. The formation of these complexes is likely to occur in areas of tissue injury and thrombosis [14]. Upon these complexes’ formation, Vn undergoes conformational changes that expose the cell adhesion domain of Vn, which can then avidly bind to integrins. Active Vn is also present on the extracellular matrix and in platelets, organized in active multimeric tissue forms [15]. Changes in Vn conformation and multimerizations are fundamental to regulate Vn functions. Nonetheless, each motif of Vn has a specific structure and organization that allows this multivalent protein to express its function and interaction with a specific biological ligand. Interestingly, in the wound-healing area, Vn is cleaved by thrombin, elastase, and plasmin, so that the protein is no longer able to bind the PAI-1 [14]. The protein switches from an antifibrinolytic protein impeding the conversion of plasminogen to plasmin, to a profibrinolytic protein, which dissociates PAI-1 and converts it into a non-inhibitory protein [13]. The binding activity of Vn can be triggered in vitro upon urea treatment or heating. These processes induce a conformational change that triggers a transition of the inactive, native form to an active form of Vn. Lastly, residues Y56 and Y59 of Vn have been determined as tyrosine sulphation sites of this protein. Sulphated tyrosines play a role in the conformation stability of Vn, as their acidic nature promotes intramolecular binding to the basic HBD3 located at the C-terminal region of Vn, stabilizing the protein in its inactive native form [16].

Vn plays several physiological functions in the host. At the level of the extracellular matrix (ECM), Vn binds all the major ECM components through its several domains, integrins, and several receptors such as proteoglycans or collagen, mediating important roles in cell adhesion and migration, as well as cell spreading and wound healing [17]. Blood Vn functions as a biological “superglue”; through binding to complement, heparin, and thrombin–antithrombin III complexes, this protein is in fact involved in the regulation of clot formation, playing a fundamental role as a key controller of mammalian tissue repair [17]. Importantly, this aspect spills over in the case of sepsis, a condition associated with the highest mortality rates for patients in intensive care units [18]. At a pathological level, the main event that triggers microvascular thrombosis and multi-organ dysfunction till death during sepsis is the uncontrolled activation of the coagulation cascade [19]. In particular, several bacterial toxins, such as the lipopolysaccharide (LPS) from Gram-negative bacteria, target the haemostasis system, altering the coagulation cascade and fibrinolytic systems, thus playing a detrimental role in the development of sepsis [19]. Interestingly, sepsis caused by Gram-negative bacteria is also associated with acute kidney injury (AKI) due to an increased renal PAI-1 activity observed in patients affected by sepsis. PAI-1, at the level of the kidneys, increases renal fibrin deposition, leading to AKI. Vn is among the causes of AKI during sepsis, as through binding to PAI-1, it traps it in its active form in the ECM [18].

From a pathological aspect, altered levels of Vn have been reported in different types of cancer, such as breast cancer or neuroblastoma, as malignant cells exploit Vn to migrate to different body districts [20,21]. Indeed, serum concentration of Vn is considered a good biomarker for tumor progression evaluation [20].

### 1.2. Vitronectin as a Regulator of the Complement System

Also known as complement cascade, the complement system is a part of the human immune system that complements the phagocytic cells in defending the host against invading microorganisms to prevent bacterial infections. The complement system is made up of several distinct plasmatic proteins that react with each other to clear bacteria from the system [22]. Once activated, the complement system leads to the release of antimicrobial compounds, complement peptides (anaphylatoxins), opsonization through C3b deposition on bacterial surfaces, and finally the formation of the terminal complement complex (TCC) or the soluble membrane attack complex (MAC) [22]. As a matter of fact, plasma from patients suffering from bacterial infections often displays increased levels of MAC biomarkers [23].

The complement system can be activated through three different pathways, namely the alternative, classical, and lectin pathways, depending on the activation induced by spontaneous or specific recognition molecules [22]. The activation of any pathway leads to the assembly of the C5b, C6, C7, C8, and C9 proteins, which form a pore in the cell membrane, leading to cell lysis [23]. However, not all cells are vulnerable to lysis by MAC deposition [24]. The thick cell wall of Gram-positive bacteria, for example, naturally protects them from MAC-mediated cell lysis, even though the sub-lytic activity of the MAC is still able to trigger signal transduction pathways [24]. Complement activation promotes the activation of an inflammatory process as well, leading to opsonization, increased phagocytosis, and the recruitment of leucocytes [25]. Upon triggering, the complement system must be tightly regulated by soluble and membrane-bound proteins in order to avoid an excessive response and subsequent self-damage to host tissues. Proteins that inhibit the complement system are the C4b-binding protein (C4BP) for the factor I, factor H, clusterin, and Vn. The latter contributes to the complement system’s regulation by binding the C5b-7 peptide at the metastable membrane-binding site, therefore inhibiting MAC insertion in the terminal pathway. Moreover, Vn can also act as a direct inhibitor of C9 polymerization [26].

### 1.3. Vitronectin as a Mediator of Cell Migration and Adhesion

Beyond the role as a regulator of the complement system, Vn mediates cell adhesion as well. Vn is anchored to the ECM via its collagen-binding or heparin-binding domains and it mediates cell adhesion, spreading, and migration via interaction with several classes of integrins (α3β1, αvβ1, αvβ3, αvβ5, and αIIbβ3) [17]. Integrins in the various host cell types are usually found in clusters at cell attachment sites, named focal adhesions [27]. Integrins binding by receptors outside of the cell internally promote the attachment and remodeling of the intracellular actin cytoskeleton, stimulating the activity of different signaling molecules [27]. Notably, the Arg-Gly-Asp (RGD) cell-binding site at the level of the N-terminal region of Vn promotes cell adhesion via Vn docking and activation of integrin receptors on the cell membrane [28]. Integrins bound by Vn trigger signaling pathways that activate different processes, such as cytoskeletal reorganization, intracellular ion transport, or gene expression [28].

## 2. Bacterial Engagement of Vitronectin as a Weapon to Escape the Immune System

The complement system promotes either direct bacterial lysis by MAC deposition or tags the pathogens to be killed by phagocytic cells. Interestingly, pathogens use related evasion strategies to counteract the complement system. Among the several evolved strategies, they all bind the human complement regulators factor H, factor H-like protein 1, and C4BP, as well as plasminogen to their surface. Moreover, many of them acquire human Vn as a very common approach to inhibit the TCC deposition and subsequent cell lysis [29].

There are two major bacterial binding regions along Vn. The great majority of bacterial proteins described to date for their Vn binding, interact with the C-terminus of the glycoprotein [30]. In particular, many microbial proteins bind Vn at the same 23 residues (352–374) [30]. Although, to a lesser extent, different bacteria recruit Vn at the level of the N-terminal or central domains [30].

**Figure 2:**
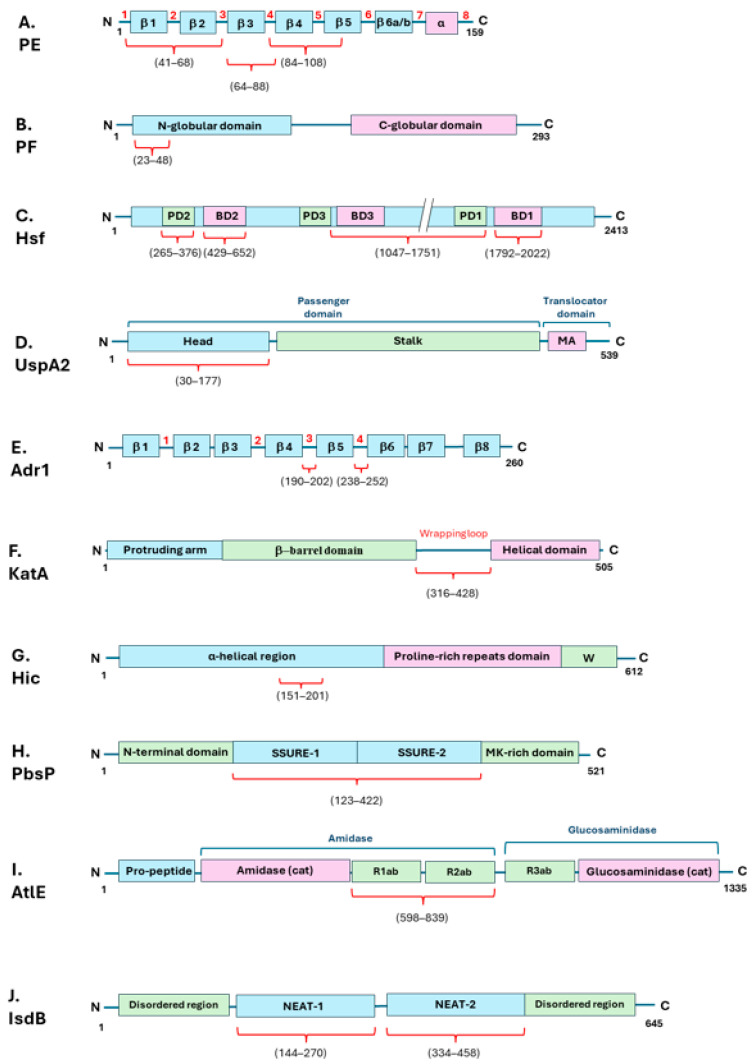
A schematic representation of bacterial vitronectin-binding proteins from Gram-positive and Gram-negative bacteria. Each protein is identified with a capital letter (**A**–**J**) and by its acronym. N- and C-terminal sites of each protein are indicated with their respective residues reported in black. Vitronectin-binding sites are indicated for each protein by red curly brackets, and respective amino acid residues are reported in round brackets. Protein loops (**A**,**E**) are indicated by red numbers. Protein domains were sourced from the UniProt Knowledgebase (UniProtKB). For further details and resources, see the references in the text. The structures reported here are representative of those for which the structural organization and Vn-binding sites have been determined.

*Haemophilus influenzae* is a Gram-negative occasional pathogen commonly carried in the upper respiratory tract of around 80% of healthy children. The Nontypeable *H. influenzae* (NTHi) can give rise to conjunctivitis, otitis media, sinusitis, and eventually pneumonia [31]. The human host is usually able to counteract NTHi infections through the complement system’s activation [32]. On the other hand, this bacterium is able to recruit Vn to its surface through binding by the surface adhesins protein E (PE) [32,33] and protein F (PF) [34]. PE is a lipoprotein present as a dimer in solution and each monomer comprises six-stranded antiparallel β-sheets linked by loops and a rigid α-helix at the C-terminus, which is tethered to the concave side of the sheet by a disulfide bridge (Figure 2A) [33]. Hallström et al. have identified three different Vn-binding sites on the PE protein: the major Vn-binding region is in the central part of the protein, spanning the four and five antiparallel β-sheets, with Lys 85 and Arg 86 localized at the level of the fourth loop, being the most important amino acid residues involved in the interaction (Figure 2A and Table 1). Two additional regions bind Vn, albeit with a lower binding affinity (Figure 2A and Table 1) [32,33,34,35]. The Vn domain involved in PE interaction is the C-terminal heparin-binding domain (HBD-3) (Figure 1 and Table 1) [33]. PF is constituted by distinct N- and C-terminal globular domains, interlinked by a long helix backbone (Figure 2B) [36]. Its Vn-binding region is located at the N-terminus of PF (Figure 2B and Table 1) [34]. PF recruits Vn at the level of the HBD-3 and the C-terminal PAI-1 binding site of the Vn molecule (Figure 1 and Table 1) [34].

The interaction of PE and PF with Vn promotes resistance to complement-mediated killing and is heparin dependent. Su et al. have observed that bacterial binding to Vn does not interfere with Vn functions; therefore the human protein retains its ability to delay MAC formation on bacterial membrane [34]. As this is a successful strategy for bacterial survival in the host, the PF protein appears to be highly conserved and detected in all clinical NTHi isolates under investigation [34]. PF promotes Vn-dependent bacterial adhesion and complement-mediated killing resistance by delaying MAC formation on the bacterial surface [34]. Among the typeable *H. influenzae* strains, the serotype F strain associated with the development of increasing invasive diseases exploits the factor H-binding protein (PH) to bind the C-terminal region of Vn and resist against complement-mediated killing (Figure 1 and Table 1) [37]. *H. influenzae* type B (Hib) expresses on its surface the *Haemophilus* surface fibril (Hsf), a representative of the major trimeric autotransporter adhesin (TAA) family. TAA is a family of adhesins that generally enable many Gram-negative pathogens to adhere to/interact with the host [38,39]. The bacterium recruits Hsf to bind Vn and therefore inhibit the terminal pathway and MAC deposition. TAAs are modular, highly repetitive proteins commonly present in the outer membrane of many Gram-negative bacterial species, which mediate adhesion to external surfaces [38]. Hsf is a multidomain molecule, which displays three epithelial cell-binding domains (BDs) and three putative domains (PDs) of unknown function (Figure 2C). Vn interaction occurs at the level of the BD2 domain and with a lower affinity also at the level of the PD2, BD1, and 1047–1751 fragment (Figure 2C and Table 1). Hsf recruits Vn at the level of the HBD3 on the C-terminal region, indeed this interaction is heparin dependent (Figure 1 and Table 1) [39].

*Moraxella catarrhalis* is another nasopharyngeal Gram-negative pathogen spread among children, which commonly causes otitis media. *M. catarrhalis* is able to subvert complement-mediated killing through C4BP and Vn recruitment through the trimeric autotransporter adhesins ubiquitous surface proteins A2 and A2H (UspA2 and UspA2H, respectively), with UspA2 being the major Vn ligand [40]. The UspA protein can be divided into a distant head, followed by a stalk and membrane-anchoring region (MA) (Figure 2D). UspA2 exploits the N-terminal head domain to target Vn at the level of the C-terminal located HBD3 (Figure 1 and Figure 2D and Table 1). Notably, only the trimeric UspA2^30–177^ can bind Vn, whereas monomeric UspA2^30–177^ does not [41]. Interestingly, the N-terminal head domain is highly diverse among the bacterial strains; nonetheless all the various variants bind Vn at the level of the C-terminal located HBD3 domain (Figure 1 and Table 1) [42].

Rickettsia is a family of Gram-negative, obligate intracellular bacteria transmitted by arthropod vectors to mammals, which cause fever and other diseases. The Rickettsia adhesin Adr1 is a surface-associated protein expressed by the species *R. conorii*, which promotes bacterial resistance to the host immune system through Vn binding. Adr1 is composed of eight transmembrane beta sheets, constituting the membrane spanning barrel, and four connecting beta strands termed “loops” that protrude into the extracellular environment (Figure 2E). It has been reported that loops three or four are sufficient to bind Vn and escape the host immune system (Figure 2E and Table 1) [43]. For this bacterial species as well, interaction with Vn resides at the level of the C-terminal domain of the human protein (Figure 1 and Table 1) [44]. Interestingly, the addition of increasing concentrations of NaCl can inhibit the Vn/Adr1 interaction, suggesting an electrostatic type of interaction. On the other hand, the presence of heparin does not affect the interaction between the two proteins. For this reason, the Adr1/Vn interaction differs from other Gram-negative bacterial/Vn interactions, as they usually are heparin dependent as it has been well documented in the literature [30].

A similar interaction mechanism as per the Rickettsia family is adopted by *Neisseria meningitidis* (Nm), the cause of meningitis and septicaemia worldwide [45]. Once in the blood stream, Nm survival relies on the ability to avoid killing by the host immune system. Among the several surface proteins already characterized for their interaction with the various complement system components, this Gram-negative bacterium is able to recruit Vn on its surface through the outer membrane protein C Opc and avoid complement-mediated killing by the inhibition of the deposition of the MAC [45]. Interestingly, this invasive bacterial species commonly displays a capsule able to mask the surface-associated proteins. Therefore, the functional efficacy of the subcapsular adhesins, such as Opc, is favored either when the bacteria are acapsulate, or in conditions of increased inflammatory cytokines circulating in the host. The latter situation promotes the expression of the cell surface receptor, that is, it is therefore able to overcome the inhibitory effects played by the capsular polysaccharides [45]. *N. meningitidis* is able to bind Vn through another molecule as well, the meningococcal surface fibril (Msf), in a heparin-independent manner. Both Opc and Msf interaction with Vn occurs at the level of the N-terminal region (Figure 1 and Table 1). Msf is a trimeric autotransporter adhesin sharing common structural architecture with Hsf from *H. influenzae* [46]. The Vn-binding region of the Msf protein is located between amino acids 39–82 of the mature protein (Table 1). Interestingly, this region can elicit an antibody response that could reduce pathogen survival within the host and therefore could be used as a potential vaccine antigen candidate [47].

*Yersiniae* and *Salmonellae* are Gram-negative bacterial species that infect humans upon the ingestion of contaminated food or water, causing gastrointestinal diseases associated with several symptoms, such as diarrhea or enterocolitis [50,51,52]. Both these species are able to bind Vn on their bacterial surface to escape complement-mediated killing. *Yersinia pestis,* the agent of plague, binds Vn through the attachment invasion locus outer membrane protein Ail [51]. Vn recruitment by the Ail protein occurs at the level of the hemopexin domains of Vn [52]. The outer membrane Protease E PgtE from *Salmonella* instead directly cleaves Vn and PAI-1 [48]. *Y. enterocolitica* adopts the Yersinia adhesin A (YadA) to interact with Vn via its C-terminal heparin-binding domain HBD-3 and is able to evade the host complement system [49]. YadA is a trimeric autotransporter adhesin structurally homologous to UspA2 from *M. catarrhalis* [74]. As for UspA2, Vn binding by YadA occurs at the level of its head domain. Interestingly, this region is able to recognize glycan moyeties; therefore YadA is able to bind glycosylated Vn (Table 1) [50].

*Helicobacter pylori* is a spiral-shaped Gram-negative colonizer of the human stomach of over half of the global population [53]. *H. pylori* binds Vn through the catalase A, KatA, acquiring complement resistance and the ability to evade the innate host immune response [53]. The primary binding site for KatA binding to Vn corresponds to the hemopexin-like domains two and three (Figure 1 and Table 1). Generally, *H. pylori* does not enter the bloodstream, nonetheless it is exposed to the complement system, since both complement factors and regulators, Vn included, are present at the level of the gastric epithelium during bacterial infection [53]. KatA structure is similar to other catalases, which are organized in tetramers. Each monomer is made of an N-terminal protruding arm, a central β-barrel domain, and a C-terminal helical domain, linked to the β-barrel one by an extended ‘wrapping’ loop (Figure 2F). KatA binding to Vn occurs at the level of the central region of the extended wrapping loop (residues 316–428) (Figure 2F and Table 1) [53].

*Pseudomonas aeruginosa* is a Gram-negative opportunistic pathogen bacterium of humans, which can cause several chronic diseases. It is also a common cause of acute lung infections in cystic fibrosis patients [54]. The Dihydrolipoamide dehydrogenase Lpd is a surface-exposed moonlighting protein, present also in the cytoplasm of *P. aeruginosa* [54]. Lpd binds several human plasma proteins and complement regulators, including Vn. Like that for many other bacterial proteins, Vn–Lpd interaction is inhibited dose dependently by increasing concentrations of heparin, as it occurs via the C-terminal heparin-binding domain (HBD) of Vn [54] (Figure 1 and Table 1). Moreover, Lpd has a second binding site on Vn, at the level of the middle region of the protein as well (Figure 1 and Table 1), suggesting the importance of this interaction for MAC inhibition [54]. Interestingly, Paullson et al. demonstrated that both *H. influenzae* and *P. aeruginosa* are able to trigger Vn production through outer membrane vesicles’ release in mice lungs and in human cell cultures. Higher levels of Vn in the bronchoalveolar space are then used by bacteria to gain protection from complement-mediated killing [75].

Among the animal colonizers, the *Riemerella anatipestifer* is a Gram-negative bacterium that elicits infections in poultry, especially in ducklings and geese, causing deep losses in industries [55]. The outer membrane protein 76, OMP76, from this bacterium has been recently described as a key escape-associated virulence factor in this important pathogen, as it is able to bind Vn (Table 1) and enhance the serosensitivity of the bacterium to a complement-mediated escape [55].

Leptospira is a genus of bacteria able to colonize the kidneys of reservoir animals and cause leptospirosis, which can also infect humans as occasional hosts. *Leptospira interrogans* displays on its surface the leptospiral complement regulator-acquiring protein A (LcpA), which is able to bind several human complement molecules simultaneously. Da Silva et al. demonstrated that this protein is also able to bind Vn at the level of the heparin-binding domains (Table 1) [56]. Given the simultaneous ability to bind different complement system proteins, LcpA might exploit Vn to evade the immune system.

*Borrelia miyamotoi* is a relapsing fever microorganism that infects humans, causing fever, headache, myalgia, arthralgia, and eventually meningitis. This bacterium displays on its surface the *Bo*rrelia miyamotoi protein BOM1093, a Vn-binding protein that contributes to serum resistance in vitro. Vn binding occurs through the C-terminal region of BOM1093 (residues 209–308) (Table 1) [58].

As Gram-positive bacteria are known to be intrinsically resistant to MAC-dependent killing, Vn recruitment to escape the TCC deposition on their surface is a strategy pursued and described most often by Gram-negative bacteria [76].

Nonetheless, the Gram-positive *Streptococcus pneumoniae* serotype 3 is also able to adopt Vn binding, together with factor H binding, to evade the immune system [62]. This bacterium is often a harmless colonizer of the human nasopharynx, which can cause several diseases of the mucosa and respiratory tract, such as sinusitis or pneumonia [62]. *S. pneumoniae* possess several approaches to fight and escape the host immune system. Among the several strategies adopted, strains expressing class I pneumococcal surface protein C proteins (PspCs) are able to recruit Vn to their surface to escape the host’s defence system [62]. In particular, the binding of the PspC-like protein factor H-binding inhibitor of complement Hic to Vn at the level of the C-terminal heparin-binding domain of Vn allows the bacterium to prevent the TCC formation during the pneumococcal infection of the host. Hic is composed of a long stretch containing regions with predicted α-helical conformation, followed by a proline-rich repeats domain and a cell wall-spanning domain (W) (Figure 2G) [77]. The binding of Hic to Vn involves the central α-helical region of Hic (Figure 2G and Table 1) [61]. Even though the Hic protein and the classical PspC proteins from the same bacterial species share only a slight sequence homology, they share a similar binding behavior, as they all evolve to bind Vn at its C-terminal HBD3 (Figure 1 and Table 1), suggesting the importance of Vn binding for this bacterial species [62]. Interestingly, increasing salt concentrations progressively inhibit the ability of the Hic to bind Vn, suggesting an electrostatic interaction between the negatively charged amino acids in the bacterial protein and Vn. This mechanism is very similar to the *R. conorii* Adr1/Vn interaction [44].

Many different respiratory pathogens recruit Vn via the third heparin-binding region at the level of the C-terminal region of the protein (Table 1). This segment represents the major binding region of the human TCC inhibitor for several microbes. This interaction, together with the benefits that the bacterium gains, identifies a common evolutionary pathway for the bacterial evasion of the host immune system, which highlights its importance for their survival.

## 3. Bacterial Targeting of Vitronectin for Host Colonization

Bacterial adhesion to host cells constitutes the first fundamental step to colonize and establish an infection. Bacterial pathogens display several factors on their surface to adhere to host surfaces, such as pili and different surface-exposed membrane or cell wall-anchored proteins, called adhesins [78,79], which recognize the different receptors present on the host cell surface. In cases of tissue damage, bacteria might gain access to the ECM and therefore bind their receptors, colonizing and eventually invading the host. Being both an ECM and plasma component, Vn is a common route adopted by both Gram-positive and Gram-negative bacteria to adhere to host cells through different adhesins/systems (Table 1). Moreover, Vn displays distinct binding sites for pathogens and epithelial cells; therefore it can constitute a molecular bridge between several pathogenic microorganisms and integrins, whose integrin-dependent signaling can promote bacterial entry into host cells [80].

### 3.1. Gram-Positive Bacteria

Streptococci are Gram-positive bacteria ubiquitously found in the human body, mostly in the urogenital and gastrointestinal tract, which can act either as harmless commensal or deadly pathogens in humans. Streptococcal interaction with Vn for host cell adhesion purposes was firstly described in 1988, when the authors described how different strains of Streptococci were able to adhere to endothelial cells from blood vessels via Vn binding [81]. Thereafter, different streptococcal adhesins have been described for their binding to Vn. In 1990, different *S. dysgalactiae* isolates from cattle with mastitis were described for their ability to adhere to bovine epithelial cells through Vn binding (Table 1) [63]. Vn promotes *S. pneumoniae* adhesion to A549 alveolar epithelial and human brain-derived microvascular endothelial cells (HBMECs). Moreover, *S. pneumoniae* is able to invade nasopharyngeal epithelial cells through the Vn-mediated interaction with αvβ3 integrins. The Vn binding of *S. pneumoniae* occurs at the level of the C-terminal heparin-binding site, as increasing concentrations of heparin are able to inhibit *S. pneumoniae* interaction with Vn. Interestingly, Bergmann et al. showed that bacterial interaction with Vn promotes cellular invasion through a dynamic actin cytoskeleton rearrangement mediated by an integrin-linked kinase activated by Vn binding to integrin αvβ3. Moreover, the authors showed that the bacterium binds preferentially multimeric forms of Vn rather than monomeric forms, indicating that this bacterium has cell-bound multimeric Vn as a favorite host target [82].

Lastly, *S. agalactiae* adopts the cell wall-anchored plasminogen binding surface protein PbsP to adhere to and invade the pulmonary and intestinal epithelial cells, by binding to Vn and exploiting the host Vn/α_v_ integrin axis. PbsP is a well-characterized GBS virulence factor containing two repeated streptococcal surface repeat (SSURE) domains following the N-terminal region, and a methionine and lysine-rich region, defined as the MK-rich region in the C-terminal region, which is involved in plasminogen binding (Figure 2H) [83]. The SSURE domains one and two are homologous to domains found in other streptococcal species and are the two domains involved in interactions with Vn (Figure 2H and Table 1) [66].

Staphylococci are colonizers of the human skin and opportunistic pathogens that can cause severe diseases, such as osteomyelitis, endocarditis, skin infections, and infections associated with indwelling medical devices [84]. Staphylococcal binding to Vn was first reported in 1987 [85,86]. According to the work of Dai-Qing et al., *Staphylococcus epidermidis* displays different proteins on its surface that are able to bind Vn, highlighting the importance of multiple recognition sites on bacterial surfaces to promote bacterial interaction with Vn and the subsequent colonization of the host. Interestingly, the various adhesins identified are expressed at different levels on the basis of the growth conditions tested (e.g., growth on blood agar or in Todd Hewitt broth) [87]. The autolysin E (AtlE) from *S. epidermidis* is a surface-associated protein that mediates attachment to polystyrene surfaces. AtlE exhibits Vn-binding activity, suggesting a function in bacterial adhesion that might involve specific interactions with plasma proteins present on polymer surfaces [67]. The full-length AtlE is processed proteolytically in vivo after the removal of the N-terminal pro-peptide into an N-terminal amidase domain, containing the amidase catalytic site (amidase cat) and two repeating regions further subdivided into a- and b-type subunits (R1_ab_ and R2_ab_), and a C-terminal glucosaminidase domain, containing a third repeat region (R3_ab_) at its N-terminal region and the glucosaminidase catalytic site (glucosaminidase cat) (Figure 2I). Both glucosaminidase and amidase are important bacterial enzymes, in which the repeating structures interact with teichoic acids and peptidoglycan to guide the enzymes toward the site of cell separation [88]. AtlE recruitment of Vn occurs at the level of the R1_ab_ and R2_ab_ repeating structures (amino acid residues 598–839) (Figure 2I and Table 1) [68,88].

*Staphylococcus aureus* cell wall-anchored adhesin iron-regulated surface determinant B (IsdB) is a receptor for host Vn. This adhesin is not constantly displayed on bacterial surfaces, but it is expressed under iron starvation conditions, as it is involved in iron uptake from the environment. IsdB displays in its central region two structurally conserved near-iron transporter (NEAT) motifs that bind haemoglobin and heme, flanked by two disordered regions at the N-terminal and C-terminal regions (Figure 2J). IsdB recruits Vn through both its subdomains NEAT1 and NEAT2 independently, and the binding occurs at the level of the heparin-binding site(s) (Figure 2J and Table 1). Interestingly, the bacterium is able to sequester Vn from plasma through IsdB and to promote bacterial adhesion and invasion of HeLa cervicovaginal epithelial cell monolayers and HUVEC endothelial cell monolayers through Vn interaction [72]. It is worth noting that recent single-molecule analyses conducted through single-molecule force spectroscopy experiments have recently revealed that IsdB binding to Vn and integrins is strongly enhanced by mechanical stress conditions that promote staphylococcal adhesion to the host [89].

*Clostridioides difficile* is a strict anaerobic Gram-positive pathogen transmitted through the fecal–oral route, which produces highly resistant spores persistent in the environment. Castro-Còrdova et al. have recently demonstrated that the spore produced by this bacterium are able to enter into the intestinal barrier via pathways dependent on host fibronectin-α5β1 and Vn-αvβ_1_. In particular, Vn binding occurs at the level of the (Bacillus ortholog) exosporium collagen-like protein BclA3, expressed on the spore surface (Table 1) [73].

### 3.2. Gram-Negative Bacteria

Gram-negative bacteria exploit Vn binding not only to escape the host complement system, but to adhere to host surfaces as well. Singh et al. observed that bacteria lacking *Haemophilus* surface fibril Hsf displayed a dramatically reduced adherence to and invasion of monolayers of the alveolar epithelial cell line A549, compared to wild-type (wt) strains in the presence of Vn. The results suggest that the Hsf fibrils may contribute to bacterial adhesion and invasion through Vn binding (Table 1) [39]. Among H- influenzae serotypes, the NTHi non-typeable one was also reported for its ability to penetrate into bronchial epithelial cells through the binding of Vn, expressed on the surface of BEAS-2B epithelial cells. Initially characterized as an extracellular pathogen, according to diverse studies, this bacterium is actually able to break into bronchial epithelial cells, probably to evade the host immune system [90].

The Ail outer membrane protein in *Y. pestis*, previously characterized for its role in complement evasion [51], has been recently reported as being involved in the invasion of Chinese Hamster Ovary (CHO) cells as well [91]. Whether this newly identified role is mediated by Ail interaction with Vn still remains to be elucidated.

The adhesion of the Gram-negative *bacillus P. aeruginosa* to the human airways is a key initial step in the establishment of infection in patients with cystic fibrosis [92]. *P. aeruginosa* adopts the αvβ5-Vn pathway to adhere to human A549 pulmonary epithelial cells, underlying the importance of Vn interaction for this bacterium not only to evade the host immune system, but for a successful colonization of the host as well [93].

*Neisseria meningitidis* Opc protein does not simply mediate the bacterium evasion of the host immune system. This protein acts as an invasion as well, as it promotes bacterial adhesion and the invasion of human brain endothelial cells through the binding of Vn at the level of residues 43–68, more precisely at the sulphated tyrosines (Table 1). Moreover, *N. meningitidis* is able to interact with Vn at the level of the second sites, i.e., the C-terminal heparin binding HBD-3 of Vn (Figure 1 and Table 1) [45].

*Mycoplasma hyorhinis* is a common pathogen of swine, which is also associated with different human tumors. There is still lack of knowledge concerning the pathogenic mechanism of this species. *M. hyorhinis* is able to adhere to swine PK-15 cells and human NCI-H292 cells through the DnaK protein. DnaK is a highly conserved protein belonging to the heat shock protein family of molecular chaperones. Recently, the DnaK protein has been characterized for its ability to interact with different ECM proteins, Vn included (Table 1) [59]. Vn–DnaK interaction still needs to be further elucidated.

## 4. Vitronectin Binding by Bacteria with a Yet-to-Be-Defined Activity

The most diverse bacterial species recruit Vn on their surface to subvert the host immune system or adhere to the host surfaces. Despite various research studies, many interactions are still unknown and need to be studied more intensively. Other than IsdB protein, which is already characterized for its involvement in bacterial adhesion and invasion of human cells through Vn binding [72], *S. aureus* displays the autolysin A (AtlA) protein that displays a high similarity both in sequence and domain organization with AtlE from *S. epidermidis* [70] and is able to bind caprine Vn. Patak et al. have recently showed that AtlA interacts with caprine Vn through a binding site that differs from the domains usually involved in bacterial adhesins’ recruitment (heparin-binding domain and the second RGD motif of goat Vn), but the exact binding domain, as well as the role in AtlA interaction with Vn, has not been identified yet (Table 1) [71].

Besides AtlE from *S. epidermidis*, this bacterium also displays another surface-exposed protein, the autolysin/adhesin Aae, which is able to bind Vn. The biological significance of this interaction has not been identified yet (Table 1) [69].

Brucellosis is a highly common bacterial zoonotic disease. Brucella display on their surface several adhesion factors, which are necessary to adhere to and invade different cell types and tissues. Among these, the *Brucella* protein Bp26 is able to bind several ECM ligands, including Vn [57]. The protein binds Vn through different N-terminal domains and through two regions located at the C-terminal domain, suggesting the importance of this interaction for the bacterium (Table 1). Nonetheless, the advantages for this interaction have not been described yet.

*Haemophilus ducreyi* is a Gram-negative pathogen causative of sexually transmitted genital ulcer diseases. Among the several proteins that confer serum resistance to this bacterium, ducreyi serum resistance A DsrA is an outer membrane protein belonging to the trimeric autotransporter adhesins family and is structurally similar to UspA2 [40]. DsrA mediates bacterial protection from host complement activity and is involved in Vn binding as well through the C-terminal region of the passenger domain of DsrA (Table 1). Despite Vn’s role as regulator of the complement system, Leduc et al. have observed that Vn binding by DsrA is not required for *H. ducreyi* serum resistance; therefore the role of DsrA binding to Vn needs to be still clarified [60].

*Streptococcus pyogenes* is a human-specific pathogen causing infections ranging from mild to severe, like necrotizing fasciitis and toxic shock syndrome. This bacterium is able to bind Vn at the level of the hemopexin-type repeats; however the adhesin involved in this interaction, as well as the role of this interaction, are still unknown (Figure 1 and Table 1) [64].

*Streptococcus suis* is an emergent zoonotic etiologic agent of septicemia, pneumonia, endocarditis, arthritis, and meningitis both in pigs and humans. During a screening regarding *S. suis* serotype 2’s ability to bind different extracellular matrix components, the bacterium’s ability to bind Vn has been reported, but the adhesin involved in this interaction has not been identified yet (Table 1) [65].

## 5. Vitronectin Adsorption on Biomaterial Surfaces: A Double-Edged Weapon

The ability of newly implanted biomaterials to adhere to cells is a critical aspect to consider during the evaluation of the biocompatibility of implants. It is fundamental to create an implant with proper physicochemical characteristics of the surface, which will promote cell affinity on the basis of the amount of adsorbed proteins from the extracellular matrix [94]. As a major component of the extracellular matrix, Vn is a crucial mediator protein to be adsorbed on the surface of biomaterials. Interestingly, a recent study from Li et al. has provided new insight into the structural evolution of Vn on material surfaces [95]. The authors observed that Vn seems to be preferably adsorbed by negatively charged surfaces rather than positively charged ones, as Vn itself is negatively charged. In particular, a negatively charged surface promotes the layering of Vn molecules, accumulating them to form a high-density protein layer. On the other hand, non-charged hydrophobic surfaces crush the Vn molecules, stacking them into a high-density multilayer by tracking adsorption [95]. Collectively, according to each substrate characteristic, the patterns for Vn adsorption can change. Charged substrates promote cell adhesion to biomaterials due to the orientation of the RGD domains on the N-terminal region of Vn. More precisely, upon interaction with the biomaterial, the SMB domain unfolds into an RGD-flexible orientation, conferring Vn a higher cell-binding capability. It is noteworthy that the Vn attachment on biomaterial surfaces may represent a double-edged sword: if, on one hand, it can promote biomaterial vascularization and cell adhesion on the implant, it will increase the biocompatibility of the implant for the host; however, on the other hand it could constitute a route for bacterial dissemination on the biomaterial, where the bacterium could potentially create biofilms and cause infections in the patient that are difficult to eradicate. Several adhesins involved in Vn binding are exploited by bacterial species to form biofilms as well. Biofilms are bacterial communities that can help microorganisms to adhere to the surfaces of medical devices, such as joint protheses, mechanical heart valves, or catheters, causing biofilm-associated infections of the bioimplants. Bacteria embedded in biofilms are difficult to eradicate and become naturally protected against antimicrobial agents [96,97]. Moreover, Hessenauer et al. recently demonstrated that Vn is able to promote the vascularization of porous polyethylene biomaterial, which is frequently used in reconstructive surgery [98]. As a consequence, the Vn-mediated intensified implant vascularization could favor the biomaterial implementation at usually unfavorable sites for implantation. Concomitantly, bacterial pathogens might use the newly created vascularization of the implant to further disseminate in the host. Therefore, Vn adsorption on biomaterial surfaces needs to be further investigated in order to modulate its functionality on advanced biomaterials and to design implants safe from risks of bacterial contamination.

## 6. Conclusions

Microorganisms have evolved several strategies to escape the host immune system and disseminate in the host. Vn is a multidomain glycoprotein that plays the most varied function in the host. As a regulator of the host complement system, Vn recruitment allows many bacterial pathogens to inhibit the MAC deposition on their cell wall, and therefore escape the complement system’s attack. In this review, we have updated the list of several surface proteins from a wide variety of microorganisms involved in this strategy. At present, the great majority of bacteria involved in Vn recruitment to escape the innate host immunity belong to Gram-negative bacteria. The reason behind this resides in the biological structure of Gram-positive bacterial cells. The Gram-positive cell wall is very thick compared to the peptidoglycan layer of Gram-negative bacteria; therefore Gram-positive cells are naturally protected from MAC-mediated cell lysis. For this reason, Vn recruitment by Gram-positive bacterial pathogens has not been deeply investigated. However, Vn recruitment by the Gram-positive *S. pneumoniae* for the prevention of MAC deposition and the subsequent escape of the host immune system during pneumococcal infection of the host has been described. Moreover, the sub-lytic activity of the MAC is still able to trigger signal transduction pathways to induce cell lysis [24]. Therefore, in view of the fact that Vn recruitment as a route to evade the complement system is commonly used by Gram-negative bacteria, but it has also been reported for a Gram-positive species, Vn recruitment by Gram-positive bacteria for the latter strategy needs further studies.

As a component of the extracellular matrix, the second main function of Vn consists of mediating cell adhesion through its RGD integrin-binding domain [6]. Because of this very reason, Vn is often exploited by bacterial pathogens as a link to adhere to and eventually invade host epithelial cells. Here, we provide a comprehensive overview of most of the bacterial molecules that behave as adhesins interacting with Vn, and some of them are also involved in bacterial internalization in host cells. The vast majority of bacterial proteins bind Vn at the level of the C-terminal region [30]. By binding different classes of integrins present on the host cell surfaces through its free N-terminal RGD motif, Vn can thus act like a physical molecular bridge for bacterial cells, promoting their adhesion and the colonization of the host. Moreover, Vn interaction with host cell integrins can initiate signal transduction pathways, which would trigger cytoskeleton remodeling and reorganization [27]. This feature can in some cases favor bacterial internalization in the host cells, which could then disseminate in the host, gaining an increased survival rate.

Recent studies indicate that Vn is well adsorbed on biomaterials used to create auxiliary aids for hospitals, or protheses implants; moreover its adsorption can promote the vascularization of the implants. This peculiar behavior of Vn could confer a successful implantation in the patient, but at the same time it could represent a novel route for bacterial dissemination on abiotic surfaces. As many bacterial pathogens tend to form biofilm on abiotic surfaces, further work is needed in this direction.

The exact mechanism of bacterial proteins/factors interactions with Vn has been widely studied but has not yet been clarified completely. Here, we report the schematic representation of the bacterial proteins involved in Vn binding characterized at present and indicate their regions involved in interactions with Vn. However, the structure of many factors that bind Vn is still missing. The high-resolution crystal structure of these proteins in their apo-form and/or in complex forms with full-length Vn or Vn domains may help to provide a deeper understanding of this interaction, and hopefully give new insights for the design of novel bacterial therapeutic agents. Many of the bacterial proteins involved in Vn interactions described here are highly conserved among bacterial strains. Conserved protein antigens expressed on the surface of bacteria constitute critical factors for the formulation of new therapies/preventive medicines against bacteria. It is also important to identify the bacterial surface proteins that interact with Vn in clinically relevant bacterial species but have not yet been assessed. In light of these considerations, it is deemed essential to investigate the molecular mechanisms underlying the interaction between bacteria and Vn and to conduct further investigations to identify new bacterial adhesins for Vn. These results will be of great support in the development of new antibacterial strategies, especially in light of the increasing number of antibiotic resistances in all Gram-positive and -negative strains. 

## Figures and Tables

**Table 1:** Bacterial factors involved in Vn interaction.

Bacterial Species	Bacterial Protein Interacting with Vn	Bacterial Protein Region Involved in Vn Binding (Amino Acid Residues)	Vn Region Bound (Amino Acid Residues)	Role in Complement Evasion	Role in Cell Adhesion/Invasion	Ref.
Gram-negative						
Nontypeable *Haemophilus influenzae*	PE	84–108, 41–68, 64–88,	HBD-3 (353–363)	+	U	[33,35]
“	PF	23–48	HBD-3 (348–361), PAI-1 binding site (348–370)	+	U	[34,36]
*Haemophilus influenzae* serotype F	PH	U	C-terminal (352–362)	+	U	[37]
*Haemophilus influenzae* serotype B	Hsf	265–376, 429–652, 1047–1751, 1792–2022	C-terminal (352–374)	+	+	[38,39]
*Moraxella catarrhalis*	UspA2	30–177	HBD-3 (312–396)	+	U	[40,41,42]
“	UspA2H	30–177	HBD-3 (312–396)	+	U	[42]
*Rickettsia conorii*	Adr1	190–202, 238–252	C-terminal (363–373)	+	U	[43,44]
*Neisseria meningititis*	Opc	U	N-terminal (43–68), HBD-3	+	+	[45]
“	Msf	39–82	N-terminal (43–68)	+	U	[46,47]
*Salmonella*	Pgte	U	C-terminal	+	U	[48]
*Yersinia enterocolitica*	YadA	Head domain	HBD-3	+	U	[49,50]
*Yersinia pestis*	Ail	U	Hemopexin domain	+	U	[51,52]
*Helicobacter pylori*	KatA	316–428	C-terminal (229–339)	+	U	[53]
*Pseudomonas aeruginosa*	LpD	U	C-terminal (354–363) Hemopexin-like repeats (161–287)	+	U	[54]
*Riemerella anatipestifer*	OMP76	U	U	+	U	[55]
*Leptospira interrogans*	LcpA	U	HBD (s)	+	U	[56]
*Brucella*	Bp26	46–65, 96–115, 146–160, 176–190, 231–250	U	U	U	[57]
*Borrelia miyamotoi*	BOM1093	209–308	U	+	U	[58]
*Mycoplasma hyorhinis*	DnaK	U	U	U	+	[59]
*Haemophilus ducreyi*	DsrA	C-terminal passenger domain	U	U	U	[60]
Gram-positive bacteria						
*Streptococcus pneumoniae*	Hic	151–201	C-terminal (HBD-3)	+	U	[61,62]
*Streptococcus dysagalactiae*	U	U	U	U	+	[63]
*Streptococcus pyogenes*	U	U	Hemopexin-type repeats	U	U	[64]
*Streptococcus suis*	U	U	U	U	U	[65]
*Streptococcus agalactiae*	PbsP	123–422	U	U	+	[66]
*Staphylococcus epidermidis*	AtlE	598–839	U	U	+	[67,68]
“	Aae	U	U	U	U	[69]
*Staphylococcus aureus*	AtlA	U	U	U	U	[70,71]
“	IsdB	144–270, 334–458	HBD (s)	U	+	[72]
*Clostridioides difficile*	BclA3	U	U	U	+	[73]

+: protein is involved in the indicated role; U: unknown.

## Data Availability

No new data were created or analyzed in this study. Data sharing is not applicable to this article.
